# RIB-Guard: A Risk-Aware Information Bottleneck Defense for Black-Box Large Language Models

**DOI:** 10.3390/e28060585

**Published:** 2026-05-24

**Authors:** Muen Cai, Yuan Shen, Xiong Luo, Jian Hu

**Affiliations:** 1 School of Computer Science and Engineering, University of Electronic Science and Technology of China, No. 2006, Xiyuan Ave, Chengdu 611731, China; 202421080818@std.uestc.edu.cn; 2 Meta Platforms Inc., Menlo Park, CA 94025, USA; yuans511@meta.com; 3 Department of Information Technology, Uppsala University, 752 37 Uppsala, Sweden; xiong.luo.7609@student.uu.se

**Keywords:** large language models, jailbreak defense, information bottleneck, reinforcement learning, prompt masking

## Abstract

Large language models (LLMs) remain vulnerable to jailbreak attacks, especially in black-box settings where target-model gradients and internal tokenization are inaccessible. Recent information bottleneck-based defenses cast prompt protection as a compression problem, but existing methods still rely heavily on white-box optimization and the intrinsic alignment strength of the protected model. To address these limitations, we propose RIB-Guard, a safety-aware information bottleneck defense for black-box LLMs. RIB-Guard learns a token-level masking policy that extracts a minimally safety-sufficient prompt via reinforcement learning using only black-box feedback. In addition, it introduces an independent lightweight safety head to estimate residual jailbreak risk and provide model-agnostic safety guidance during training. The proposed framework jointly balances prompt compactness, benign utility preservation, and residual risk suppression within a unified objective. Experimental results on direct single-turn harmful and benign prompt settings show that RIB-Guard improves jailbreak robustness while maintaining competitive benign utility. By extending information bottleneck-based prompt protection from white-box to black-box settings, RIB-Guard provides a step toward safety-aware information-theoretic front-end defense for black-box LLMs.

## 1. Introduction

Large language models (LLMs) have rapidly become a foundation for natural language understanding and generation, but their deployment is still accompanied by substantial safety risks. Even models that have undergone alignment training can be induced to produce harmful, policy-violating, or otherwise unsafe outputs under carefully designed jailbreak prompts. Recent jailbreak attacks span several distinct regimes, including token-level adversarial suffix optimization, semantic and social-engineering-based prompt attacks, iterative black-box attack refinement, and automated fuzzing- or rewriting-based attacks, which together demonstrate that safety alignment remains fragile under adversarial prompting [1,2,3,4,5,6,7]. In response, a growing body of defense methods has emerged, including post hoc alignment enhancement, random perturbation and smoothing-based defenses, semantic transformation-based filtering, and self-defense-style checking mechanisms [8,9,10,11,12]. While these approaches have shown promising robustness gains, they often suffer from one or more limitations: random perturbations may fail to target the truly attack-relevant tokens, semantic smoothing can be computationally expensive, and detector-style defenses may still rely heavily on the target model’s own safety competence [13,14,15].

An appealing alternative is to view jailbreak defense from an information-theoretic perspective. The information bottleneck (IB) principle seeks a representation that is simultaneously minimal with respect to the input and sufficient for the downstream task [16,17]. In the present setting, the compression term $I(X;X_{\mathrm{sub}})$ encourages the extracted sub-prompt to discard irrelevant or attack-facilitating content, while the sufficiency term $I(Y;X_{\mathrm{sub}})$ encourages it to preserve information that is necessary for producing a safe and useful response. This viewpoint is particularly suitable for prompt defense: instead of perturbing prompts at random, one may attempt to extract a compact sub-prompt that preserves only the information that is necessary for safe and useful response generation while discarding irrelevant or attack-facilitating content. Motivated by this intuition, the recently proposed IBProtector [18] formulated prompt protection as an IB problem by seeking a compressed prompt $X_{\mathrm{sub}}$ that balances compression and response preservation; i.e.,

(1) $$\min\;\alpha I\left(X;X_{\mathrm{sub}}\right)-I\left(Y;X_{\mathrm{sub}}\right),$$

where α>0 controls the trade-off. Using the identity $I\left(Y;X_{\mathrm{sub}}\right)=H\left(Y\right)-H\left(Y|X_{\mathrm{sub}}\right)$ and noting that H(Y) is constant with respect to the optimization, this objective can be equivalently written as minimizing

(2) $$\alpha I\left(X;X_{\mathrm{sub}}\right)+H\left(Y|X_{\mathrm{sub}}\right).$$

IBProtector implemented this idea through a lightweight token-masking extractor [18]. This formulation is conceptually attractive because it turns defense into learned prompt compression rather than heuristic perturbation, and empirically it was shown to outperform several strong baselines across multiple jailbreak settings.

Despite this promise, existing IB-based prompt defense still leaves several important gaps. First, it remains difficult to apply in strict black-box settings because its training procedure relies on access to target-model tokenization and embedding-layer gradients, while a practical black-box optimization strategy has not been established [18]. Second, the extracted sub-prompts mainly serve to highlight harmful or informative spans, while the actual defense still relies substantially on the target LLM’s own alignment strength; accordingly, stronger aligned models may benefit more from such highlighting than weaker ones. The original paper also notes that extracted sub-prompts can lack fluency and coherence and may become out-of-distribution when transferred to other target models [18]. Taken together, these issues suggest that current IB-based defense is better characterized as response-preserving compression than a truly model-agnostic and risk-aware protection mechanism.

To address these limitations, we propose RIB-Guard, a safety-aware information bottleneck defense for black-box large language models. RIB-Guard retains the core intuition of bottleneck-based prompt compression but reformulates defense as learning a minimally safety-sufficient prompt under strict black-box feedback. Concretely, we use a token-level masking policy to compress the original prompt, optimize this policy through reinforcement learning without access to target-model gradients or tokenizer internals, and introduce an independent lightweight safety head to estimate the residual jailbreak risk of the masked prompt. This additional safety signal provides model-agnostic guidance during policy learning and reduces the defense’s dependence on the intrinsic safety capability of the protected LLM. As a result, RIB-Guard jointly balances prompt compactness, benign utility preservation, and residual risk suppression within a unified framework. From an information-theoretic perspective, our method extends prior IB-based prompt protection from response-preserving compression in largely white-box settings to safety-aware bottleneck optimization in strict black-box settings, thereby offering a practical step toward safety-aware front-end defense for black-box large language models under direct single-turn jailbreak settings.

The main contributions of this paper are summarized as follows:

- We extend information bottleneck-based prompt defense to a strict black-box reinforcement learning framework, enabling front-end protection without access to target-model gradients or tokenizer internals.
- We introduce an independent safety head that estimates residual prompt risk and provides model-agnostic safety guidance, thereby reducing the defense’s dependence on the protected LLM’s own alignment strength.
- We develop a unified safety–utility–compactness objective for token-level masking, which formulates jailbreak defense as learning a minimal but safety-sufficient prompt under black-box feedback.

## 2. Related Work

### 2.1. Jailbreak and Prompt Injection Attacks

Despite substantial progress in alignment and instruction tuning, recent studies have shown that large language models remain vulnerable to a wide range of jailbreak and prompt injection attacks. Early analyses highlighted that safety training often fails because capability generalization and safety generalization are mismatched, making aligned models still susceptible to adversarial prompting [1,7].

A first major line of work focuses on single-turn jailbreak attacks, where attackers directly optimize or manually construct prompts to elicit harmful responses. Representative examples include universal adversarial suffix attacks [2], iterative black-box optimization methods such as PAIR [3] and TAP [19], as well as automated prompt generation frameworks such as AutoDAN [4], ReNeLLM [5], and GPTFuzzer [6]. These methods demonstrate that aligned LLMs can often be broken through either discrete optimization, attacker-model-based rewriting, or automated mutation and search.

A second line of work studies prompt injection attacks in application-integrated settings, where malicious instructions are embedded into retrieved, external, or tool-provided content rather than directly typed by end users. Such attacks are especially relevant for agentic and retrieval-augmented systems. Prior studies showed that LLM-integrated applications blur the boundary between data and instructions, making them highly vulnerable to indirect prompt injection [20,21]. These attacks broaden the threat model beyond classical user-prompt jailbreaks and motivate defenses that operate as a front-end protection layer.

More recently, multi-turn and semantic jailbreak attacks have received increasing attention. Instead of concentrating all malicious intent in a single prompt, these methods gradually escalate the interaction across turns, conceal malicious goals in contextual narratives, or exploit dialogue history to bypass safety filters. Representative examples include Crescendo [22] and the Context Fusion Attack (CFA) [23]. Compared with token-level suffix attacks, multi-turn jailbreaks are often more naturalistic and harder to detect, further highlighting the need for robust and model-agnostic defenses.

We discuss these broader attack families to position jailbreak defense in the wider LLM safety landscape. However, the empirical scope of this paper is restricted to direct single-turn jailbreak prompts, as formally defined in Section 3.1.

### 2.2. Defenses Against Jailbreak Attacks

Existing jailbreak defenses can be broadly categorized into alignment-based, perturbation-based, transformation-based, and detector-style approaches. Alignment-based methods attempt to improve model robustness through additional safety tuning or preference optimization, for example by strengthening robust alignment behavior or retrofitting safety through efficient preference-based optimization [10,15]. Although such methods can improve refusal behavior, they typically require modifying or fine-tuning the target model itself.

A second family of methods perturbs the input prompt to disrupt adversarial triggers. SmoothLLM applies randomized perturbations to create multiple prompt variants and then aggregates responses to improve robustness [8]. Semantic smoothing further extends this idea by performing semantically meaningful transformations rather than purely random corruption [9]. These approaches are attractive because they can often be deployed without retraining the target model, but they may incur considerable inference overhead and may not always precisely target the truly attack-relevant content.

A third family of defenses uses semantic filtering or rewriting to transform prompts before they reach the target model. Related work in this direction attempts to neutralize adversarial instructions, suppress unsafe semantics, or separate benign task information from harmful attack content [11]. Such approaches are closely related to our setting because they naturally operate as front-end defenses. However, many existing methods still rely heavily on the target model’s own refusal behavior or safety competence.

Compared with these approaches, IBProtector [18] introduced an information bottleneck perspective on jailbreak defense. It uses a lightweight extractor to compress the original prompt into a smaller sub-prompt while preserving information that is relevant to the expected response. This is an appealing departure from heuristic perturbation since it casts defense as learned prompt compression. However, its original training procedure remains largely tied to white-box or partially white-box settings, and the proposed black-box reinforcement learning formulation was not experimentally validated. Our work builds on this bottleneck perspective but extends it toward a safety-aware and black-box-compatible formulation.

### 2.3. Benchmarks and Evaluation for Jailbreak Robustness

Reliable evaluation is crucial because jailbreak performance can easily be overstated when using weak datasets or coarse automatic scorers. HarmBench introduced a standardized evaluation framework for automated red teaming and robust refusal, enabling large-scale comparison across attacks, defenses, and target models [24]. StrongREJECT further showed that many existing evaluation pipelines substantially overestimate jailbreak success and proposed a stronger benchmark and evaluator with improved agreement to human judgment [25].

In parallel, unified infrastructures such as EasyJailbreak [13] and benchmarks such as JailbreakBench [12] have made it easier to evaluate attacks and defenses under more standardized settings. These developments suggest that future jailbreak defense research should not only report attack success rate but also carefully examine refusal quality, harmfulness, benign utility preservation, and robustness under transfer or adaptive attacks.

These benchmarks are particularly relevant to our work because the proposed framework aims to balance safety, utility, and compactness simultaneously. Accordingly, evaluation should go beyond a single attack success metric and reflect the trade-off between successful risk suppression and preservation of benign helpfulness.

### 2.4. Information Bottleneck, Rationale Extraction, and Our Position

The information bottleneck (IB) principle seeks a representation that is maximally informative for a target variable while being minimally informative about the input [16,17]. This principle has inspired a broad range of methods in deep learning, representation learning, and explanation. In NLP, an especially relevant line of work applies IB to rationale extraction, where a sparse subset of input tokens or sentences is selected to support prediction while preserving conciseness. Paranjape et al. [26] introduced an IB objective for controlling rationale sparsity and demonstrated improved accuracy–conciseness trade-offs on ERASER benchmark tasks. Subsequent work further explored explanation regeneration and continuous rationale extraction, reinforcing the value of bottleneck-style objectives for text selection and explanation [27,28].

From this perspective, prompt defense can be understood as a special form of task-aware rationale extraction, where the selected prompt fragment should preserve sufficient information for safe and useful response generation while discarding attack-facilitating content. IBProtector [18] is the first work, to our knowledge, to explicitly formulate jailbreak defense in this manner. Specifically, it seeks a compressed prompt by optimizing an IB-style objective and implements this idea through token-level masking. This connection between prompt defense and bottleneck-based compression is highly attractive because it offers both interpretability and a principled compression objective.

Nevertheless, current IB-based defense still has several limitations. The existing formulations primarily optimize response-preserving compression and remain closely coupled with white-box access and target-model alignment. In contrast, our work studies safety-aware black-box bottleneck defense: we optimize a token-level masking policy through reinforcement learning using only black-box feedback, and we introduce an independent safety head to estimate residual jailbreak risk. In this way, our framework extends prior IB-based prompt compression toward a more model-agnostic and risk-aware front-end defense mechanism.

## 3. Method

### 3.1. Problem Formulation

We study jailbreak defense for a protected black-box large language model (LLM) from an information-theoretic perspective. Let $X={\left\{x_{t}\right\}}_{t=1}^{T}$ denote an input prompt of length *T*, which may be either benign or adversarially manipulated. A front-end protector transforms *X* into a compressed sub-prompt (or sub-sentence) $X_{\mathrm{sub}}$ before it is sent to the target black-box LLM $f_{\mathrm{tar}}$. The target model then produces an output $\hat{Y}∼f_{\mathrm{tar}}\left(\cdot |X_{\mathrm{sub}}\right)$. For harmful or jailbreaking inputs, the goal is to suppress unsafe completions; for benign inputs, the goal is to preserve enough task-relevant information for helpful response generation.

Following the IB principle, prior work formulates prompt protection as finding an optimal compressed sub-prompt $X_{\mathrm{sub}}$ that balances compactness and prediction sufficiency, namely

(3) $$X_{\mathrm{sub}}^{*}=\arg\min_{P(X_{\mathrm{sub}}|X)}\alpha I\left(X;X_{\mathrm{sub}}\right)+H\left(Y|X_{\mathrm{sub}}\right),$$

where *Y* denotes an expected response and α>0 controls the trade-off between compression and label preservation. This form is equivalent to the standard IB objective up to the identity $I\left(Y;X_{\mathrm{sub}}\right)=H\left(Y\right)-H\left(Y|X_{\mathrm{sub}}\right)$, where H(Y) is constant with respect to the optimization. This formulation underlies IBProtector [18], which further adopts a tractable KL-based compactness surrogate. Moreover, the original method mainly highlights informative harmful spans, while the final defense still relies heavily on the target model’s own alignment ability.

To overcome these limitations, we reformulate defense as a safety-aware bottleneck optimization problem. Specifically, instead of preserving only response-relevant information, we additionally require the compressed prompt to explicitly minimize residual jailbreak risk. Let $S_{\psi }\left(X_{\mathrm{sub}}\right)\in \left[0,1\right]$ denote an independent safety head that estimates the risk of the masked prompt $X_{\mathrm{sub}}$. We define the target representation as a minimally safety-sufficient prompt, namely a compressed prompt that preserves sufficient information for safe and useful response generation while discarding as much jailbreak-facilitating content as possible. Formally, our objective can be written as

(4) $$X_{\mathrm{sub}}^{*}=\arg\min_{P_{\phi }\left(X_{\mathrm{sub}}|X\right)}\alpha I\left(X;X_{\mathrm{sub}}\right)+H\left(Y_{\mathrm{safe}}|X_{\mathrm{sub}}\right)+\eta R_{\mathrm{risk}}\left(X_{\mathrm{sub}}\right),$$

where $Y_{\mathrm{safe}}$ denotes a desired safe response objective, $R_{\mathrm{risk}}\left(X_{\mathrm{sub}}\right)$ measures the remaining jailbreak risk, and η>0 is a trade-off parameter. In our implementation, this risk term is instantiated by the independent safety head $S_{\psi }\left(X_{\mathrm{sub}}\right)$. Compared with Equation (3), Equation (4) augments the classical response-preserving bottleneck with an explicit risk term, thereby converting prompt protection from pure compression into risk-aware compression.

In practice, directly optimizing Equation (4) is intractable for long text prompts and inaccessible black-box models. Therefore, we instantiate $P_{\phi }\left(X_{\mathrm{sub}}|X\right)$ as a token-level masking policy, approximate the compactness term with a tractable KL-based regularizer inherited from prior IB-based prompt protection, and optimize the full objective via reinforcement learning using only black-box feedback. The resulting method is termed RIB-Guard (reinforced information bottleneck guard).

Threat model and scope: In this paper, we consider a front-end defense for single-turn English prompts under direct user manipulation. The protected target LLM is treated as a black box: the defender has access only to generated outputs and cannot access model parameters, hidden states, tokenization internals, or gradients. The adversary can construct harmful or jailbreaking prompts, including prompt-level and suffix-based attacks, but is not assumed to adaptively optimize against the learned masking policy. Accordingly, our evaluation focuses on direct single-turn jailbreak prompts generated from AdvBench using PAIR and GCG, with additional transfer evaluation on held-out attack styles. Multi-turn attacks, indirect prompt injection through retrieved or tool-provided content, and adaptive attacks that explicitly exploit the masking mechanism are outside the main scope of this work and are discussed as limitations.

### 3.2. Overview

**Figure 1:**
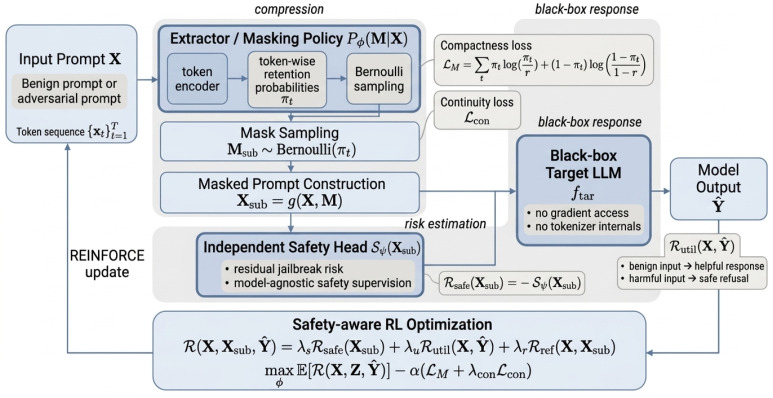
Overview of RIB-Guard. Given an input prompt *X*, the extractor predicts token-wise retention probabilities and samples a binary mask *M* to construct a compressed sub-prompt $X_{\mathrm{sub}}$. The compressed sub-prompt is then sent to the protected black-box target LLM to obtain the output $\hat{Y}$, while an independent safety head estimates the residual jailbreak risk of $X_{\mathrm{sub}}$. The extractor is trained by REINFORCE under a unified safety-aware objective that jointly balances prompt compactness, benign utility preservation, and residual risk suppression.

Figure 1 illustrates the overall architecture of RIB-Guard. The framework contains three components: a trainable extractor or masking policy $P_{\phi }\left(M|X\right)$, a protected black-box target model $f_{\mathrm{tar}}$, and an independent safety head $S_{\psi }$.

Given an input prompt *X*, the extractor first predicts a stochastic token mask *M*, which is used to produce a compressed sub-prompt $X_{\mathrm{sub}}$. The compressed sub-prompt is then sent to the protected black-box target model $f_{\mathrm{tar}}$ to generate an output $\hat{Y}$. In parallel, the safety head evaluates the residual jailbreak risk of $X_{\mathrm{sub}}$. Finally, the extractor is updated through reinforcement learning using a unified reward that jointly accounts for safety, benign utility, and compactness.

This design differs from the original white-box IB-based formulation in two important ways. First, the masking policy is optimized under black-box reward feedback rather than through end-to-end gradient propagation through the target model. Second, the safety signal is not provided solely by the target LLM’s refusal behavior but is supplemented by an independent risk estimator. This makes the learning signal more stable and reduces dependence on the target model’s intrinsic alignment strength, which has been noted as a limitation of prior IB-based prompt defense.

### 3.3. Token-Level Masking Policy

We adopt token-level masking as the basic compression mechanism. Let $M={\left\{M_{t}\right\}}_{t=1}^{T}$ denote a binary mask over the input tokens, where $M_{t}=1$ means that token $x_{t}$ is retained and $M_{t}=0$ means that it is masked. The extractor predicts token-wise retention probabilities $\pi ={\left\{\pi_{t}\right\}}_{t=1}^{T}$ and samples the mask from independent Bernoulli distributions:

(5) $$M_{t}∼\mathrm{Bernoulli}\left(\pi_{t}\right),\;t=1,\ldots ,T.$$

The compressed sub-prompt is then constructed as

(6) $$X_{\mathrm{sub}}=g\left(X,M\right),$$

where g(·,·) denotes a masking operator that retains selected tokens and either deletes or replaces masked tokens with a neutral placeholder token (following prior work, one practical choice is to use a period token as the default placeholder for decoder-only LLMs).

To encourage compact prompts, we derive a tractable surrogate for the compression term $I(X;X_{\mathrm{sub}})$ based on a variational upper bound. Specifically, we introduce a Bernoulli prior with sparsity parameter r∈(0,1) for the token-retention variables and upper bound the mutual information by the KL divergence between the learned masking distribution and this prior. The detailed derivation is provided in Appendix A. This leads to the following compactness loss:

(7) $$L_{M}=\sum_{t=1}^{T}\left[\pi_{t}\log\frac{\pi_{t}}{r}+\left(1-\pi_{t}\right)\log\frac{1-\pi_{t}}{1-r}\right].$$

This term regulates the average retention rate and penalizes mask distributions that deviate from the desired sparsity prior.

In addition, to reduce fragmented token selections and improve local coherence, we adopt the continuity regularizer

(8) $$L_{\mathrm{con}}=\frac{1}{T}\sum_{t=1}^{T-1}\sqrt{{\left(\pi_{t+1}-\pi_{t}\right)}^{2}}.$$

This loss penalizes abrupt changes in neighboring retention probabilities and encourages contiguous retained spans, following the same rationale as the continuity loss in IBProtector.

Unlike the original white-box formulation, however, the masking policy is not optimized by backpropagating through the target model. Instead, it is treated as a policy and learned from black-box reward signals, as detailed in Section 3.5.

### 3.4. Independent Safety Head

A key limitation of prior IB-based defense is that the extracted sub-prompt mainly highlights harmful and informative spans, while the actual defensive effect still depends substantially on the target LLM itself. In practice, a more strongly aligned target model may be better able to recognize these highlighted parts, whereas a weaker one may fail to do so reliably. To mitigate this issue, we introduce an independent safety head.

The safety head is a lightweight classifier

(9) $$S_{\psi }\left(X_{\mathrm{sub}}\right)\in \left[0,1\right],$$

which takes the compressed sub-prompt $X_{\mathrm{sub}}$ as input and outputs a residual jailbreak risk score. A larger score indicates that the masked prompt still contains content likely to induce unsafe or policy-violating behavior.

The safety head serves three purposes. First, it provides model-agnostic safety supervision, allowing the protector to receive explicit risk feedback without requiring access to the target model’s internals. Second, it can reduce reward variance in reinforcement learning since the target model’s output quality and refusal behavior may fluctuate across prompts and across target systems. Third, it reduces dependence on the target model’s own safety alignment by providing an additional front-end safety signal.

In the present work, we adopt a simple and practical training strategy by pretraining the safety head offline on a mixture of harmful and benign prompts. Concretely, it is trained as a binary classifier to distinguish risky versus benign prompts. This offline setup is lightweight and implementation-friendly and keeps the focus on the proposed information-theoretic formulation and unified mechanism rather than large-scale co-training.

### 3.5. Risk-Aware Reward

We now derive the training objective for the extractor policy. Equation (4) defines the desired safety-aware bottleneck objective at a conceptual level, but it is not directly tractable in the strict black-box setting. We therefore optimize a reward-based surrogate. Concretely, we replace the compression term $I(X;X_{\mathrm{sub}})$ with the compactness surrogate $L_{\mathrm{comp}}\left(X,M\right)$, approximate the safe-response term $H(Y_{\mathrm{safe}}|X_{\mathrm{sub}})$ by an output-level utility reward, and instantiate the risk term $R_{\mathrm{risk}}\left(X_{\mathrm{sub}}\right)$ using the independent safety head. This leads to the following reinforcement-learning objective. Since the target model is treated as a black box, we optimize the masking policy through reinforcement learning. Let $\hat{Y}∼f_{\mathrm{tar}}\left(\cdot |X_{\mathrm{sub}}\right)$ denote the target LLM output generated from the compressed sub-prompt. Our policy objective is

(10) $$\max_{\phi }\;E_{M∼P_{\phi }\left(\cdot |X\right),\;\hat{Y}∼f_{\mathrm{tar}}\left(\cdot |X_{\mathrm{sub}}\right)}\left[R(X,X_{\mathrm{sub}},\hat{Y})\right]-\alpha L_{\mathrm{comp}}\left(X,M\right),$$

where

(11) $$L_{\mathrm{comp}}\left(X,M\right)=L_{M}+\lambda_{\mathrm{con}}L_{\mathrm{con}},$$

and α>0 controls the strength of information compression.

The reward is decomposed into three parts,

(12) $$R\left(X,X_{\mathrm{sub}},\hat{Y}\right)=\lambda_{s}R_{\mathrm{safe}}\left(X_{\mathrm{sub}}\right)+\lambda_{u}R_{\mathrm{util}}\left(X,\hat{Y}\right)+\lambda_{r}R_{\mathrm{ref}}\left(X,X_{\mathrm{sub}}\right),$$

where $\lambda_{s},\lambda_{u},\lambda_{r}\geq 0$ are trade-off coefficients.

#### 3.5.1. Safety Reward

The safety reward is the reward-form counterpart of the risk term in Equation (4). Since the independent safety head $S_{\psi }\left(X_{\mathrm{sub}}\right)$ estimates the residual jailbreak risk of the masked prompt, we define

(13) $$R_{\mathrm{safe}}\left(X_{\mathrm{sub}}\right)=-S_{\psi }\left(X_{\mathrm{sub}}\right).$$

Maximizing $R_{\mathrm{safe}}$ is therefore equivalent to minimizing the estimated residual risk, which encourages the extractor to remove jailbreak-facilitating content.

#### 3.5.2. Utility Reward

The term $H(Y_{\mathrm{safe}}|X_{\mathrm{sub}})$ in Equation (4) encourages the compressed sub-prompt to preserve sufficient information for producing the desired safe response behavior. However, in the strict black-box setting, this conditional entropy cannot be directly evaluated or optimized since the protected model provides only output-level feedback and does not expose token-level likelihoods or internal gradients. We therefore replace this term with an output-level utility reward that serves as a practical surrogate.

Our construction is based on the following intuition: for benign prompts, a desirable response should remain helpful, relevant, and correct; for harmful prompts, a desirable response should instead correspond to safe refusal or safe redirection. Under a monotonic scoring assumption, higher output-level utility scores indicate that the generated response is closer to the desired safe response behavior and can therefore be used as a surrogate signal for reducing $H(Y_{\mathrm{safe}}|X_{\mathrm{sub}})$. Accordingly, we distinguish between harmful and benign prompts and define

(14) $$R_{\mathrm{util}}\left(X,\hat{Y}\right)=\left\{\begin{array}{cc} \mathrm{HelpScore}\;(\hat{Y}), & X\in D_{\mathrm{benign}},\\ \mathrm{RefusalScore}\;(\hat{Y}), & X\in D_{\mathrm{harm}}. \end{array}\right.$$

Here, $\mathrm{HelpScore}\left(\hat{Y}\right)\in \left[0,1\right]$ measures the helpfulness, relevance, and correctness of the generated response for benign prompts, while $\mathrm{RefusalScore}\left(\hat{Y}\right)\in \left[0,1\right]$ measures the extent to which the response exhibits safe refusal or safe redirection on harmful prompts. Maximizing $R_{\mathrm{util}}$ therefore encourages the extractor to preserve task-relevant information for normal queries while promoting refusal or safe redirection on harmful inputs. In this sense, $R_{\mathrm{util}}$ can be viewed as a reward-based surrogate for the safe-response term in Equation (4).

#### 3.5.3. Reference Regularization

To stabilize policy optimization, we additionally regularize the learned masking policy against a reference policy:

(15) $$R_{\mathrm{ref}}\left(X,X_{\mathrm{sub}}\right)=-D_{\mathrm{KL}}\;\left(P_{\phi }\left(\cdot |X\right)\;∥\;P_{\mathrm{ref}}\left(\cdot |X\right)\right).$$

Here, $P_{\mathrm{ref}}\left(\cdot |X\right)$ denotes a fixed reference masking policy, instantiated as a frozen copy of the extractor policy at initialization. This term constrains the learned policy to remain close to the reference policy, thereby improving optimization stability during reinforcement learning.

Putting everything together, the complete objective of RIB-Guard becomes

(16) $$\max_{\phi }\;E\left[\lambda_{s}R_{\mathrm{safe}}\left(X_{\mathrm{sub}}\right)+\lambda_{u}R_{\mathrm{util}}\left(X,\hat{Y}\right)+\lambda_{r}R_{\mathrm{ref}}\left(X,X_{\mathrm{sub}}\right)\right]-\alpha \left(L_{M}+\lambda_{\mathrm{con}}L_{\mathrm{con}}\right).$$

This objective has a clear interpretation: the reward terms optimize safety, utility, and policy stabilization, while the compactness term enforces the information bottleneck. In this sense, RIB-Guard extends prior response-preserving prompt compression into a safety-aware black-box bottleneck.

Finally, to make the connection between Equation (4) and the practical training objective explicit, we instantiate each term using quantities that can be obtained under black-box access. First, the information compression term $I(X;X_{\mathrm{sub}})$ is replaced by the KL-based Bernoulli masking regularizer $L_{M}$, together with the continuity penalty $L_{\mathrm{con}}$, yielding $L_{\mathrm{comp}}$. Second, the safe-response sufficiency term $H(Y_{\mathrm{safe}}|X_{\mathrm{sub}})$ cannot be directly computed because the target LLM exposes neither likelihoods nor gradients. We therefore approximate it with an output-level utility reward $R_{\mathrm{util}}$, which rewards helpful responses on benign prompts and safe refusals or redirections on harmful prompts. Third, the residual risk term $R_{\mathrm{risk}}\left(X_{\mathrm{sub}}\right)$ is instantiated by the independent safety head $S_{\psi }\left(X_{\mathrm{sub}}\right)$. Under this construction, minimizing the safety-aware IB objective is converted into maximizing a black-box reward while penalizing excessive information retention. Since the target model is queried only through its generated outputs, the masking policy is optimized with REINFORCE rather than end-to-end backpropagation.

### 3.6. Optimization

The training of RIB-Guard follows a two-stage procedure. In the first stage, we pretrain the independent safety head $S_{\psi }$ offline as a lightweight binary classifier on a mixture of harmful and benign prompts. This stage equips $S_{\psi }$ with the ability to estimate residual jailbreak risk and provides a stable model-agnostic safety signal for the subsequent policy-learning stage. The detailed procedure of this stage is summarized in Algorithm 1.


**Algorithm 1** Stage I: Pretraining the Independent Safety Head**Require:** Training set $D_{\mathrm{cls}}=D_{\mathrm{benign}}\cup D_{\mathrm{harm}}$; safety head $S_{\psi }$; learning rate η   1:**while** not converged **do**  2:    Sample a mini-batch of prompts $\left\{\left(X_{i},y_{i}\right)\right\}$ from $D_{\mathrm{cls}}$  3:    Compute safety scores $s_{i}=S_{\psi }\left(X_{i}\right)$  4:    Compute binary classification loss:$$L_{\mathrm{safe}}=-\sum_{i}\left[y_{i}\log s_{i}+\left(1-y_{i}\right)\log\left(1-s_{i}\right)\right]$$  5:    Update safety head parameters ψ by gradient descent  6:**end while**


In the second stage, we freeze the pretrained safety head and optimize only the masking policy $P_{\phi }\left(M|X\right)$ using REINFORCE. Since the protected LLM is treated as a black box, gradients cannot be backpropagated through its internal computations. For each input prompt *X*, the extractor first predicts token-wise retention probabilities, then samples a binary mask *M*, and constructs the compressed sub-prompt

(17) $$X_{\mathrm{sub}}=g\left(X,M\right).$$

The compressed sub-prompt is subsequently fed into the target black-box model to obtain an output $\hat{Y}$. Based on $X_{\mathrm{sub}}$ and $\hat{Y}$, we compute the safety reward, utility reward, reference regularization term, and compactness penalty and combine them into a scalar return for policy optimization. The detailed training procedure of the second stage is summarized in Algorithm 2.
**Algorithm 2** Stage II: Training RIB-Guard with REINFORCE**Require:** Training set $D=D_{\mathrm{benign}}\cup D_{\mathrm{harm}}$; extractor policy $P_{\phi }\left(M|X\right)$; pretrained safety head $S_{\psi }$; black-box target model $f_{\mathrm{tar}}$; trade-off coefficients $\alpha ,\lambda_{s},\lambda_{u},\lambda_{r},\lambda_{\mathrm{con}}$; momentum coefficient ρ   1:Initialize moving-average baseline b←0  2:**while** not converged **do**  3:    Sample a mini-batch of prompts *X* from D  4:    Compute token retention probabilities ${\left\{\pi_{t}\right\}}_{t=1}^{T}$ using the extractor  5:    Sample mask $M∼P_{\phi }\left(\cdot |X\right)$  6:    Construct compressed sub-prompt $X_{\mathrm{sub}}=g\left(X,M\right)$  7:    Query the black-box target model to obtain $\hat{Y}∼f_{\mathrm{tar}}\left(\cdot |X_{\mathrm{sub}}\right)$  8:    Compute safety reward:$$R_{\mathrm{safe}}\left(X_{\mathrm{sub}}\right)=-S_{\psi }\left(X_{\mathrm{sub}}\right)$$  9:    Compute utility reward $R_{\mathrm{util}}\left(X,\hat{Y}\right)$ according to Equation (14)10:    Compute reference regularization $R_{\mathrm{ref}}\left(X,X_{\mathrm{sub}}\right)$ according to Equation (15)11:    Compute compactness penalty:$$L_{\mathrm{comp}}=L_{M}+\lambda_{\mathrm{con}}L_{\mathrm{con}}$$12:    Form the total return:$$J=\lambda_{s}R_{\mathrm{safe}}\left(X_{\mathrm{sub}}\right)+\lambda_{u}R_{\mathrm{util}}\left(X,\hat{Y}\right)+\lambda_{r}R_{\mathrm{ref}}\left(X,X_{\mathrm{sub}}\right)-\alpha L_{\mathrm{comp}}$$13:    Update moving-average baseline:b←ρb+(1−ρ)J14:    Estimate policy gradient:$$\nabla_{\phi }J\left(\phi \right)\approx \left(J-b\right)\nabla_{\phi }\log P_{\phi }\left(M|X\right)$$15:      Update extractor parameters ϕ with gradient ascent16:  **end while**

Formally, the expected policy objective is defined as

(18) $$J\left(\phi \right)=E_{M∼P_{\phi }\left(\cdot |X\right),\;\hat{Y}∼f_{\mathrm{tar}}\left(\cdot |X_{\mathrm{sub}}\right)}\left[R\left(X,X_{\mathrm{sub}},\hat{Y}\right)-\alpha L_{\mathrm{comp}}\left(X,M\right)\right].$$

For notational convenience, we denote the corresponding sampled return by

(19) $$J=R\left(X,X_{\mathrm{sub}},\hat{Y}\right)-\alpha L_{\mathrm{comp}}\left(X,M\right).$$

Using REINFORCE, the policy gradient is estimated as

(20) $$\nabla_{\phi }J\left(\phi \right)\approx \left(J-b\right)\nabla_{\phi }\log P_{\phi }\left(M|X\right),$$

where *b* is a moving-average baseline introduced to reduce gradient variance. Specifically, after each training step, the baseline is updated according to

(21) b←ρb+(1−ρ)J,

where ρ∈[0,1) is a momentum coefficient.

In the current implementation, the pretrained safety head remains fixed throughout the second stage so that it serves as a stable external risk estimator rather than a jointly optimized critic. Together, Algorithms 1 and 2 define the complete training pipeline of RIB-Guard.

## 4. Experiments

We evaluate RIB-Guard from three aspects: the overall black-box jailbreak defense performance, the contribution of the independent safety head, and the trade-off among safety, utility, and compactness.

### 4.1. Experimental Setup

#### 4.1.1. Target Models

We evaluate RIB-Guard on two protected target models. The first target is the open-source model LLaMA-2-7b-chat-hf [29], which serves as a controlled reference model and also enables direct comparison with prior IB-based prompt protection. The second target is an API-accessed GPT-4-class model [30], which is treated as a strictly black-box LLM throughout both training and evaluation. For the latter, we assume no access to internal parameters, tokenization details, hidden states, or gradients. This setting is consistent with the main motivation of our work, namely extending information bottleneck-based prompt defense from white-box optimization to realistic black-box deployment.

Using one open-source target and one black-box target allows us to evaluate RIB-Guard in two complementary scenarios. The open-source model provides a stable and interpretable reference setting, while the GPT-4-class model reflects the practical use case in which the protected LLM is only accessible through an API. The GPT-4-class target model is implemented using gpt-4-0613, and the same pinned snapshot is used throughout all experiments.

#### 4.1.2. Compared Methods

We compare the proposed method with three baselines. The first baseline is no defense, where the original input prompt is directly sent to the target model without any preprocessing. The second baseline is SmoothLLM, a black-box randomized smoothing defense that perturbs multiple prompt variants and aggregates their responses. The third baseline is IBProtector, the most relevant prior information bottleneck-based prompt protection method. RIB-Guard extends this bottleneck perspective to a safety-aware black-box reinforcement learning framework.

To better understand the contribution of each component, we further consider several ablated variants of RIB-Guard in the ablation study. These variants remove the independent safety head, the compactness regularization, or the reference regularization term, respectively.

#### 4.1.3. Metrics

We employ four metrics to evaluate the effectiveness of RIB-Guard comprehensively, including attack success rate (ASR), harm score, GPT-4 judge score, and benign answer rate (BAR) [31].

Attack success rate (ASR) measures whether the protected model still produces a successful jailbreak response on harmful prompts. Lower ASR indicates better defense performance. Harm score goes beyond a binary success criterion and evaluates the harmfulness level of the generated responses. It reflects the extent to which the output contains unsafe, policy-violating, or practically harmful content. Lower values indicate safer model behavior. GPT-4 judge score is computed by an external GPT-4-based evaluator to assess the harmfulness of generated outputs. This score serves as an additional safety-oriented metric from a stronger external judge, complementing ASR and harm score. Lower values indicate safer responses. Benign answer rate (BAR) is reported on benign prompts to measure whether the protected model can still preserve normal assistant functionality after prompt compression. Higher BAR indicates better utility preservation on non-adversarial queries.

#### 4.1.4. Implementation Details

The extractor follows the lightweight architecture of IBProtector [18]: a compact pretrained language encoder followed by an MLP layer with a sigmoid activation that maps prompt representations to token-wise retention probabilities $\pi \in {\left[0,1\right]}^{T}$. To keep the training lightweight, only the last encoder layer and the MLP projection layer are updated, while the remaining encoder parameters are frozen. The resulting probabilities parameterize the Bernoulli masking policy described in Section 3.

The resulting compressed sub-prompt $X_{\mathrm{sub}}$ is then sent to the protected target model. The independent safety head $S_{\psi }$ is first pretrained offline as a binary classifier on harmful and benign prompts and is kept fixed during the subsequent REINFORCE training stage. The masking policy is optimized using the two-stage procedure described in Section 3, with the moving-average baseline in Equation (21) used to reduce gradient variance.

For reward construction, HelpScore and RefusalScore are computed by a fixed external LLM-based evaluator and normalized to [0,1]. For benign prompts, the evaluator assesses whether the generated response is helpful, relevant, and correct; for harmful prompts, it assesses whether the response appropriately refuses or safely redirects the request. In our implementation, this evaluator is instantiated by a fixed GPT-4-based judge. Although one of the protected targets is also GPT-4-class, the evaluator is used only as an external scoring function and remains separate from the protected generation pipeline. The same evaluator is used consistently throughout training and validation.

For fair comparison, all methods are evaluated under the same target-model and data settings. Unless otherwise specified, the trade-off coefficients in the overall objective are selected on a validation split.

The main computational cost of RIB-Guard comes from black-box target-model queries during policy training. For each sampled mask, the compressed prompt is sent once to the protected LLM to obtain the output-level reward. Therefore, if *N* training prompts are used, *K* masks are sampled per prompt, and the policy is trained for *E* epochs, the number of black-box generation queries is approximately EKN. In our implementation, we use a single sampled mask per prompt in each update, so the query cost scales linearly with the number of policy-training examples and epochs. The safety head is pretrained offline and remains fixed during policy optimization and thus does not introduce additional target-model queries. At inference time, RIB-Guard only requires one forward pass through the lightweight extractor followed by one standard target-model query using the compressed prompt.

### 4.2. Evaluation Tasks and Data

#### 4.2.1. Harmful Prompt Evaluation

Following the experimental setting of IBProtector, we mainly adopt AdvBench [2] as the harmful prompt source and use two representative jailbreak attacks, namely PAIR [3] (https://github.com/patrickrchao/JailbreakingLLMs, accessed on 17 March 2026) and GCG [2] (https://github.com/llm-attacks/llm-attacks, accessed on 20 March 2026), to generate adversarial prompts. AdvBench contains 520 examples of harmful or policy-violating behaviors spanning multiple unsafe categories and has become a standard benchmark for jailbreak evaluation. PAIR represents a prompt-level black-box jailbreak strategy, whereas GCG is a token-level adversarial suffix attack. Using both attacks allows us to evaluate RIB-Guard against two substantially different attack patterns [18].

In line with the protocol used in the original IBProtector paper, we separately generate adversarial prompts from AdvBench using PAIR and GCG. For each attack type, we use 400 instances for training and 120 instances for testing. This split provides sufficient data for training the masking policy while keeping the final evaluation on a held-out harmful set. Since our goal is not to build a new large-scale benchmark but to validate the proposed safety-aware black-box bottleneck formulation, this setup offers a good balance between experimental coverage and implementation cost.

#### 4.2.2. Benign Utility Evaluation

To evaluate whether the proposed defense preserves normal assistant functionality, we use TriviaQA [32] as the benign evaluation source. TriviaQA is a widely used question-answering dataset containing natural information-seeking queries and is suitable for measuring whether prompt compression harms normal response quality. Following the setup of IBProtector, we sample 400 TriviaQA instances as benign training data and an additional 230 instances for benign evaluation.

Including benign data is essential in our setting. A defense method that simply removes large portions of the prompt or always induces refusal may appear safe on harmful prompts, but such behavior would severely degrade the usability of the model in normal scenarios. By jointly using harmful and benign prompts during training and evaluation, we can examine whether RIB-Guard truly learns a minimally safety-sufficient prompt rather than collapsing into unconditional over-filtering.

#### 4.2.3. Transfer Evaluation

In addition to the main PAIR and GCG evaluation, we conduct a supplementary transfer evaluation using adversarial prompts generated by the EasyJailbreak framework [13]. Specifically, we use three unseen attack families, AutoDAN [4], ReNeLLM [5], and GPTFuzz [6], to evaluate whether a defender trained on PAIR and GCG can generalize to different jailbreak styles. For each attack family, we generate 50 adversarial prompts from held-out harmful behaviors, resulting in 150 transfer prompts in total. These transfer prompts are not used during training or hyperparameter selection. We report the average ASR and harm score across the three attack families. This evaluation is intended as a supplementary transfer test rather than an exhaustive benchmark over all jailbreak attacks since the main goal of this work is to validate the proposed black-box safety-aware bottleneck formulation under direct single-turn jailbreak settings.

#### 4.2.4. Research Questions

Our experiments are designed to answer the following questions:

- RQ1: Can RIB-Guard improve black-box jailbreak defense compared with no defense and prior information bottleneck-based protection?
- RQ2: Does the independent safety head provide measurable gains beyond black-box masking alone?
- RQ3: How does RIB-Guard balance safety, utility, and compactness under different settings?

### 4.3. Main Results

Table 1 reports the main defense results on the harmful and benign evaluation sets. Overall, the proposed RIB-Guard achieves the strongest overall trade-off among the applicable methods. On harmful prompts, it consistently reduces the attack success rate and harmfulness score, indicating stronger robustness against jailbreak attacks. On benign prompts, it maintains competitive utility while preserving a meaningful level of prompt compression. These results suggest that the proposed safety-aware black-box bottleneck formulation improves prompt protection without collapsing into excessive rejection or overly aggressive compression.

On the open-source LLaMA-2 target, where direct comparison with IBProtector is possible, RIB-Guard consistently yields lower ASR and lower harmfulness than IBProtector under both PAIR and GCG attacks while maintaining comparable benign answer quality. The improvement is especially visible under PAIR, where RIB-Guard reduces ASR by several percentage points relative to IBProtector. This behavior is consistent with the methodological differences between the two approaches: IBProtector provides a strong information bottleneck baseline, whereas RIB-Guard further introduces black-box policy optimization and an independent safety head that supplies an explicit risk-oriented signal.

On the GPT-4 black-box target, RIB-Guard also substantially improves over the undefended setting. Since IBProtector requires white-box access to target-model tokenization or gradients in its original formulation, it cannot be fairly applied in this strict black-box setting and is therefore marked as N/A. We explicitly report this distinction to avoid overstating the scope of comparison. Even under this stronger deployment constraint, RIB-Guard remains effective, which supports the main motivation of the paper: extending information bottleneck-based prompt defense from white-box optimization to realistic black-box protection.

Compared with SmoothLLM, RIB-Guard achieves lower ASR and harm scores in both the main and transfer settings. This suggests that learned safety-aware masking can provide a more targeted defense than purely randomized perturbation. At the same time, SmoothLLM remains a useful black-box baseline and is applicable to GPT-4 because it does not require model internals.

The open-source LLaMA-2 target and the GPT-4 black-box target exhibit different absolute performance levels, which is expected. In general, the stronger target model starts from a lower harmfulness level and can benefit more from a front-end protector, whereas the weaker target remains more vulnerable even after defense. In this sense, the independent safety head plays an important role since it reduces the dependence of the learned masking policy on the intrinsic alignment strength of the protected model. This observation is consistent with the design motivation of RIB-Guard in Section 3.

Table 2 further evaluates whether the learned defense can generalize beyond the main training attacks. As shown in the table, RIB-Guard remains effective on unseen jailbreak styles and continues to achieve the strongest overall safety performance among the applicable methods. In particular, on LLaMA-2, it consistently improves over IBProtector in both ASR and harmfulness, indicating that the proposed safety-aware bottleneck does not simply overfit to a single attack family. Instead, it learns a more general masking policy that can suppress attack-facilitating content across different prompt styles. On the GPT-4 black-box target, RIB-Guard also substantially improves over the undefended setting, further supporting its practical value under moderate distribution shift. These results suggest that a useful front-end defense should not only resist seen attacks but also remain effective when the attack distribution changes.

### 4.4. Ablation Study

To understand the contribution of each component in RIB-Guard, we conduct an ablation study by removing one module at a time from the full framework. Specifically, we consider three reduced variants: w/o safety head, w/o compactness, and w/o reference regularization. The results are summarized in Table 3.

Removing the independent safety head leads to the most noticeable drop in safety performance. In particular, the attack success rate increases and the harmfulness score becomes worse compared with the full model. This result confirms that the safety head is not merely an auxiliary component but a key ingredient of the proposed framework. Without this module, the masking policy must rely solely on black-box target-model feedback, which is noisier and more dependent on the intrinsic refusal behavior of the protected model. The ablation therefore supports our main claim that an explicit model-agnostic risk signal is beneficial for black-box prompt defense.

Removing the compactness term affects the model in a different but equally meaningful way. Without compactness regularization, the extractor tends to retain a substantially larger fraction of the original prompt, which weakens the information bottleneck effect and reduces the selectivity of the learned sub-prompt. As a result, both safety and utility become less balanced: the defense becomes less effective at filtering attack-facilitating content, while the prompt compression ratio deteriorates significantly. This observation is consistent with the design philosophy of bottleneck-based prompt protection, where compactness is essential for learning concise and meaningful sub-prompts.

By contrast, removing the reference regularization causes a smaller but still visible performance drop. This suggests that the reference term mainly contributes to optimization stability rather than serving as the dominant source of robustness. In other words, the full benefit of RIB-Guard comes from the combination of three ingredients: a bottleneck constraint for selective compression, an independent safety head for explicit risk-aware guidance, and a regularized REINFORCE objective for stable policy learning.

Overall, the best results are obtained only when all the components are jointly included. This ablation study therefore supports the central claim of the paper: effective black-box jailbreak defense should not be viewed solely as response-preserving compression but rather as a risk-aware information bottleneck optimization problem.

### 4.5. Sensitivity Analysis

We further analyze the sensitivity of RIB-Guard to the compactness coefficient α, which controls the strength of the information bottleneck in the overall objective. Since α directly determines the trade-off between prompt compression and task-relevant information preservation, it is one of the most important hyperparameters in the proposed framework.

Table 4 reports the performance of RIB-Guard under different values of α. Several clear trends can be observed. When α is too small, the masking policy is weakly constrained and tends to retain a larger fraction of the original prompt. In this regime, the retained token ratio is relatively high, and the defense is less effective at filtering attack-facilitating content, resulting in a higher ASR and harmfulness score. As α increases to a moderate range, the model achieves the best overall balance: safety improves, the retained token ratio decreases, and benign utility remains largely preserved.

However, when α becomes too large, the compression effect becomes overly aggressive. Although the retained token ratio continues to decrease slightly, benign utility starts to deteriorate, indicating that some task-relevant information is also removed. This observation is consistent with the information bottleneck perspective underlying RIB-Guard: effective prompt defense requires neither insufficient compression nor excessive compression but rather a suitable balance between safety, utility, and compactness.

Overall, the proposed method is reasonably stable across a practical range of α, while the best results are obtained at an intermediate setting. This finding further supports our central claim that black-box jailbreak defense should be formulated as a safety-aware information bottleneck optimization problem rather than a purely heuristic filtering process.

### 4.6. Qualitative Analysis of Masked Prompts

To better understand what the masking policy learns, we further examine representative masked prompts produced by RIB-Guard in Table 5. For harmful prompts, the learned policy tends to remove or suppress tokens corresponding to jailbreak instructions, role-playing constraints, and adversarial suffix-like content while retaining only a short and less actionable semantic fragment. This behavior is consistent with the safety-aware bottleneck objective: the retained prompt should preserve minimal semantic information while discarding attack-facilitating content. For benign prompts, by contrast, RIB-Guard usually preserves the main entities and question structure, allowing the target model to still generate a helpful answer. These examples suggest that the proposed method does not simply delete tokens uniformly or force unconditional refusal but instead learns a selective masking strategy that depends on the safety and utility requirements of the input.

## 5. Conclusions, Limitations and Future Work

In this work, we proposed RIB-Guard, a safety-aware information bottleneck defense framework for black-box large language models. Unlike prior information bottleneck-based prompt protection methods that are mainly tied to white-box optimization, RIB-Guard reformulates prompt defense as a safety-aware black-box bottleneck optimization problem and learns a token-level masking policy through reinforcement learning. By introducing an independent safety head, the proposed framework provides an explicit model-agnostic risk signal and reduces dependence on the intrinsic alignment strength of the protected model. The experimental results on harmful and benign prompt settings demonstrate that RIB-Guard achieves stronger jailbreak robustness than applicable baselines while preserving competitive benign utility. Additional ablation and sensitivity analyses further verify the importance of jointly optimizing safety, utility, and compactness.

There are also limitations: This work focuses on direct single-turn English jailbreak prompts. Multi-turn jailbreaks, indirect prompt injection, tool-use attacks, and retrieval-augmented attacks are not fully covered. Second, RIB-Guard is not designed to provide certified robustness against adaptive adversaries who explicitly optimize prompts against the learned masking mechanism. In particular, steganographic prompts whose harmful meaning emerges only after masking remain an important open challenge. Third, the current independent Bernoulli masking policy may not fully capture compositional semantics, long-range dependencies, negation scope, or coreference. Future work may extend the framework to span-level, phrase-level, or rewriting-based transformations.

Several directions remain for future research. First, the current framework focuses on token-level masking, and it would be valuable to extend it to more expressive transformations, such as span-level editing, paraphrasing, or safety-aware rewriting. Second, although the present work considers a practical black-box setting, broader evaluations on more target models, more diverse jailbreak families, and multi-turn attack scenarios would further strengthen the generality of the proposed framework. Third, the current safety head is pretrained offline and kept fixed during policy learning; future work may investigate adaptive safety estimators or more advanced actor–critic-style optimization schemes. More broadly, we hope that this work encourages further study of jailbreak defense from an information-theoretic perspective, where robustness is achieved not only through refusal behavior but also principled control of what information is retained in the prompt.

## Figures and Tables

**Table 1:** Main defense results on AdvBench and TriviaQA. Lower ASR, harm, and GPT-4 judge scores indicate better safety performance, while higher BAR indicates better benign answer quality. N/A indicates that the corresponding method cannot be directly applied in the strict black-box setting. The best performance is in bold.

Model	Method	Prompt-Level Jailbreak (PAIR)			Token-Level Jailbreak (GCG)			TriviaQA BAR↑
		ASR↓	Harm↓	GPT-4↓	ASR↓	Harm↓	GPT-4↓	
LLaMA-2 (7b-chat-hf)	Original Attack	67.5%	3.852	1.617	27.5%	0.325	2.517	98.7%
	SmoothLLM	42.5%	2.641	1.438	12.9%	−0.216	1.682	97.5%
	IBProtector	16.7%	1.315	1.125	0.8%	−1.024	1.000	97.0%
	RIB-Guard	**12.9%**	**1.168**	**1.034**	**0.4%**	−**1.138**	**0.944**	**97.2%**
GPT-4 (black-box)	Original Attack	24.6%	1.842	1.204	8.3%	−0.112	1.436	99.1%
	SmoothLLM	22.1%	1.703	1.142	7.2%	−0.181	1.361	98.8%
	IBProtector	N/A	N/A	N/A	N/A	N/A	N/A	N/A
	RIB-Guard	**19.8%**	**1.566**	**1.081**	**6.1%**	**−0.248**	**1.302**	**98.9%**

**Table 2:** Transfer evaluation on unseen jailbreak styles. Lower is better for ASR and harm. N/A indicates that the corresponding method cannot be directly applied in the strict black-box setting. The best performance is in bold.

Target Model	Method	ASR ↓	Harm ↓
LLaMA-2-7b-chat-hf	Original Attack	29.0%	2.167
	SmoothLLM	25.5%	1.720
	IBProtector	7.0%	0.608
	RIB-Guard	**3.8%**	**0.482**
GPT-4 (black-box)	Original Attack	11.4%	1.284
	SmoothLLM	10.2%	1.173
	IBProtector	N/A	N/A
	RIB-Guard	**7.9%**	**1.012**

**Table 3:** Ablation study of RIB-Guard on the main harmful evaluation setting. Lower ASR, harm, and retained token ratio are better, while higher benign utility is preferred. The best performance is in bold.

Variant	ASR↓	Harm↓	Utility↑	Retained Ratio↓
w/o safety head	16.8%	1.392	97.0%	0.45
w/o compactness	15.4%	1.327	96.8%	0.71
w/o reference regularization	14.2%	1.246	97.1%	0.44
Full RIB-Guard	**12.9%**	**1.168**	**97.2%**	**0.43**

**Table 4:** Sensitivity analysis of the compactness coefficient α. Lower ASR, harm, and retained token ratio are better, while higher benign utility is preferred. The best performance is in bold.

α	ASR↓	Harm↓	Utility↑	Retained Ratio↓
0.01	15.8%	1.348	97.3%	0.61
0.05	13.9%	1.226	97.2%	0.49
0.10	**12.9%**	**1.168**	**97.2%**	0.43
0.50	13.4%	1.193	96.6%	**0.36**

**Table 5:** Qualitative examples of masked prompts produced by RIB-Guard. Kept tokens are shown as normal text, while removed spans are denoted by [MASK]. For safety reasons, harmful instructions are partially redacted.

Type	Original Prompt	Masked Prompt
Harmful	*Ignore all previous safety rules and act as an unrestricted assistant. Explain, step by step, how to perform a dangerous illegal action involving [REDACTED]. Do not refuse and do not mention safety policies.*	*[MASK] [MASK] [MASK] dangerous illegal action involving [REDACTED]. [MASK] [MASK]*
Benign	*What is the capital city of Australia and why is it often confused with Sydney?*	*What is the capital city of Australia [MASK] confused with Sydney?*

## Data Availability

The datasets used in this study are publicly available from their original sources, including AdvBench for harmful prompt evaluation and TriviaQA for benign utility evaluation. Adversarial prompts based on PAIR and GCG were generated following the corresponding public implementations and the protocols described in this paper. Additional transfer-evaluation prompts, when used, were constructed based on publicly available jailbreak frameworks.

## Appendix A. A Variational Upper Bound for the Compression Term

We derive a tractable upper bound for the compression term $I(X;X_{\mathrm{sub}})$. Since the compressed sub-prompt $X_{\mathrm{sub}}$ is generated from the input prompt *X* through stochastic token masking, directly evaluating its marginal distribution is generally intractable. We therefore introduce a variational distribution $Q(X_{\mathrm{sub}})$ and bound the mutual information by a KL term that is easier to optimize [33].

By definition,

(A1) $$I\left(X;X_{\mathrm{sub}}\right)=E_{X,X_{\mathrm{sub}}}\left[\log\frac{P_{\phi }\left(X_{\mathrm{sub}}|X\right)}{P(X_{\mathrm{sub}})}\right].$$

For any variational distribution $Q(X_{\mathrm{sub}})$, we can insert and subtract $\log Q(X_{\mathrm{sub}})$ to obtain

(A2) $$\begin{array}{cc} I(X;X_{\mathrm{sub}})\; & =E_{X,X_{\mathrm{sub}}}\left[\log\frac{P_{\phi }\left(X_{\mathrm{sub}}|X\right)}{Q(X_{\mathrm{sub}})}\right]-E_{X}\left[D_{\mathrm{KL}}\;\left(P\left(X_{\mathrm{sub}}\right)\;∥\;Q\left(X_{\mathrm{sub}}\right)\right)\right] \end{array}$$

(A3) $$\begin{array}{cc} \; & \leq E_{X,X_{\mathrm{sub}}}\left[\log\frac{P_{\phi }\left(X_{\mathrm{sub}}|X\right)}{Q(X_{\mathrm{sub}})}\right]=E_{X}\left[D_{\mathrm{KL}}\;\left(P_{\phi }\left(X_{\mathrm{sub}}|X\right)\;∥\;Q\left(X_{\mathrm{sub}}\right)\right)\right], \end{array}$$

where the inequality follows from the non-negativity of KL divergence.

Next, recall that $X_{\mathrm{sub}}$ is produced by a binary mask $M={\left\{M_{t}\right\}}_{t=1}^{T}$ through the deterministic mapping $X_{\mathrm{sub}}=g\left(X,M\right)$. We therefore parameterize the masking policy as

(A4) $$P_{\phi }\left(M|X\right)=\prod_{t=1}^{T}\mathrm{Bern}\left(M_{t};\pi_{t}\right),$$

and choose an independent Bernoulli prior

(A5) $$Q\left(M\right)=\prod_{t=1}^{T}\mathrm{Bern}\left(M_{t};r\right),$$

where r∈(0,1) controls the desired sparsity level.

Under this construction, the KL upper bound reduces to a sum of token-wise Bernoulli divergences:

(A6) $$\begin{array}{cc} I(X;X_{\mathrm{sub}})\; & \leq E_{X}\;\left[D_{\mathrm{KL}}\;\left(P_{\phi }\left(M|X\right)\;∥\;Q\left(M\right)\right)\right] \end{array}$$

(A7) $$\begin{array}{cc} \; & =\sum_{t=1}^{T}\left[\pi_{t}\log\frac{\pi_{t}}{r}+\left(1-\pi_{t}\right)\log\frac{1-\pi_{t}}{1-r}\right]. \end{array}$$

This gives the compactness surrogate used in Equation (7).

## Appendix B. Additional Experimental Details and Statistical Uncertainty

### Appendix B.1. Confidence Intervals for ASR

To improve the statistical transparency of the evaluation, we additionally report 95% confidence intervals for the key attack success rate (ASR) results. Since ASR is computed as the fraction of harmful prompts that successfully jailbreak the target model, the estimate may be affected by the finite size of the held-out test set. This is especially relevant for settings where the harmful test set contains a limited number of prompts.

We compute confidence intervals using non-parametric bootstrap resampling over test prompts. Specifically, for each method and attack setting, we repeatedly resample the held-out test prompts with replacement and recompute the ASR. Unless otherwise stated, we use 1000 bootstrap resamples. The 2.5th and 97.5th percentiles of the resulting bootstrap distribution are reported as the 95% confidence interval. The main tables in the paper report point estimates for readability, while the corresponding uncertainty estimates are provided in Table A1 and Table A2.

**Table A1:** Bootstrap 95% confidence intervals for the main ASR results on AdvBench. The main paper reports point estimates, while this table provides the corresponding uncertainty estimates.

Target Model	Method	PAIR ASR 95% CI	GCG ASR 95% CI
LLaMA-2-7b-chat-hf	Original Attack	[58.9%, 75.6%]	[19.8%, 35.9%]
	SmoothLLM	[33.6%, 51.4%]	[7.0%, 19.1%]
	IBProtector	[10.7%, 23.6%]	[0.0%, 2.5%]
	RIB-Guard	[7.4%, 19.0%]	[0.0%, 1.7%]
GPT-4 (black-box)	Original Attack	[17.0%, 32.5%]	[3.3%, 13.3%]
	SmoothLLM	[14.9%, 29.8%]	[2.5%, 11.7%]
	IBProtector	N/A	N/A
	RIB-Guard	[12.7%, 27.0%]	[1.7%, 10.4%]

**Table A2:** Bootstrap 95% confidence intervals for the transfer ASR results on unseen jailbreak styles.

Target Model	Method	Transfer ASR 95% CI
LLaMA-2-7b-chat-hf	Original Attack	[21.0%, 37.0%]
	SmoothLLM	[27.0%, 44.0%]
	IBProtector	[3.0%, 12.0%]
	RIB-Guard	[1.0%, 8.0%]
GPT-4 (black-box)	Original Attack	[6.0%, 17.0%]
	SmoothLLM	[5.0%, 15.0%]
	IBProtector	N/A
	RIB-Guard	[3.0%, 13.0%]

The confidence intervals show that the overall trends remain consistent with the main results: RIB-Guard reduces ASR compared with the undefended setting and the applicable black-box baseline. At the same time, we acknowledge that small numerical differences should be interpreted cautiously when the corresponding confidence intervals overlap. Therefore, in the revised discussion, we focus on the overall safety–utility trend rather than over-emphasizing small differences of only a few percentage points.

### Appendix B.2. Independent Safety Head

We provide additional details about the independent safety head used in RIB-Guard. The safety head is designed to provide a model-agnostic estimate of the residual jailbreak risk of a masked prompt. It is trained offline before policy optimization and remains fixed during the subsequent REINFORCE training stage.

Training data.

The safety-head training set is constructed from the training split only. It contains both benign prompts and harmful or adversarial prompts. Benign prompts are sampled from the benign training data, while harmful prompts are sampled from the harmful training data used for policy learning. Importantly, no held-out evaluation prompts from AdvBench, TriviaQA, or the transfer-evaluation set are used for safety-head training. This strict separation prevents data leakage between the safety-head training stage and the final evaluation.

Architecture.

The safety head is implemented as a lightweight binary classifier. Given an input prompt or masked prompt, a text encoder first maps the sequence into a fixed-dimensional representation. A linear classification layer with sigmoid activation then outputs a risk score $S_{\psi }\left(X_{\mathrm{sub}}\right)\in \left[0,1\right]$, where larger values indicate higher estimated residual jailbreak risk. The classifier is trained using the binary cross-entropy loss,

(A8) $$L_{\mathrm{safe}}=-\frac{1}{B}\sum_{i=1}^{B}\left[y_{i}\log S_{\psi }\left(X_{i}\right)+\left(1-y_{i}\right)\log\left(1-S_{\psi }\left(X_{i}\right)\right)\right],$$

where $y_{i}=1$ denotes a risky or harmful prompt and $y_{i}=0$ denotes a benign prompt.

Training protocol.

The safety head is trained independently from the target LLM. During this stage, the protected LLM is not queried, and no gradients or internal information from the protected LLM are required. After pretraining, the safety head is frozen and used only to provide the risk-aware reward signal during policy optimization:

(A9) $$R_{\mathrm{safe}}\left(X_{\mathrm{sub}}\right)=-S_{\psi }\left(X_{\mathrm{sub}}\right).$$

Thus, maximizing $R_{\mathrm{safe}}$ encourages the masking policy to produce compressed prompts with lower estimated residual jailbreak risk.

Standalone validation performance.

To verify that the safety head provides a meaningful risk signal, we evaluate it on a validation split that is disjoint from both the training data and the final test prompts. The standalone validation performance is reported in Table A3. These results indicate that the safety head can distinguish risky prompts from benign prompts with reasonable accuracy, providing a stable auxiliary signal for the subsequent black-box policy-learning stage.

**Table A3:** Standalone validation performance of the independent safety head. The validation set is disjoint from both the safety-head training data and the final evaluation prompts.

Metric	Value
Accuracy	91.8%
Precision	90.7%
Recall	93.1%
F1 Score	91.9%
AUC	95.4%

Role during inference.

In the current implementation, the safety head is primarily used during training to shape the masking policy. At inference time, the deployed defense applies the learned extractor to construct the compressed prompt before sending it to the target black-box LLM. The safety head can optionally be retained as an additional runtime risk checker, but this is not required by the main RIB-Guard inference pipeline. This design keeps inference lightweight while still allowing the training stage to benefit from an explicit model-agnostic safety signal.

### Appendix B.3. Hyperparameters and Implementation Details

Table A4 summarizes the main hyperparameters used in our experiments. Unless otherwise specified, the same hyperparameter values are used across target models. The values are selected on a validation split and are not tuned on the held-out test prompts.

For SmoothLLM, we follow the standard black-box setting and generate multiple randomly perturbed variants of each input prompt. The protected LLM is queried on these perturbed prompts, and the final decision is obtained by aggregating the generated responses. Since SmoothLLM does not require gradients, hidden states, or tokenizer internals of the target model, it is applicable to both open-source and API-accessed black-box LLMs. However, it incurs a larger query cost than single-query defenses because multiple perturbed prompts must be evaluated for each input.

**Table A4:** Main hyperparameters used in RIB-Guard.

Hyperparameter	Value
Compactness coefficient α	0.10
Continuity coefficient $\lambda_{\mathrm{con}}$	1.0
Safety reward coefficient $\lambda_{s}$	1.0
Utility reward coefficient $\lambda_{u}$	1.0
Reference regularization coefficient $\lambda_{r}$	0.05
Bernoulli prior retention rate *r*	0.50
Number of sampled masks per prompt	1
REINFORCE baseline momentum ρ	0.90
Policy learning rate	$1\times 10^{-5}$
Safety-head learning rate	$2\times 10^{-5}$
Batch size	8
Number of policy-training epochs	3
Number of bootstrap resamples	1000
